# Youth in Residential Care: A Cross-Sectional Mediation Analysis of Youth’s Perceptions of Their Social Images, Self-Representations, and Adjustment Outcomes

**DOI:** 10.3389/fpsyg.2021.744088

**Published:** 2021-11-24

**Authors:** Maria Manuela Calheiros, Carla Sofia Silva, Joana Nunes Patrício, Helena Carvalho

**Affiliations:** ^1^CICPSI, Faculdade de Psicologia, Universidade de Lisboa, Lisboa, Portugal; ^2^Centro de Investigação e Intervenção Social (CIS-Iscte), Instituto Universitário de Lisboa (ISCTE-IUL), Lisboa, Portugal; ^3^Caminhos da Infância, Lisboa, Portugal; ^4^Centro de Investigação e Estudos de Sociologia (CIES-Iscte), Instituto Universitário de Lisboa (ISCTE-IUL), Lisboa, Portugal

**Keywords:** young people, residential care, youth’s perceptions of their social images, self-representations, mental health

## Abstract

Individuals’ perceptions of their social images [i.e., meta-representations (MR)] and perceived stereotyping threat create involuntary stress responses that may affect important outcomes, such as self-esteem, academic achievement, and mental health. This study aimed to (1) analyze the indirect associations between residential care youth’s MR and their psychological adjustment (i.e., externalizing and internalizing problems) through their self-representations (SR) and (2) test the moderating role of youth’s age and residential unit size in those associations. A sample of 926 youth aged between 12 and 25years old filled out self-report questionnaires regarding their representations about how people in general perceive them (i.e., MR) and their SR. Residential care professionals filled in the socio-demographic questionnaires and the Child Behavior Checklist. Data were analyzed through multiple mediation models and moderated mediation models. Results showed that (1) youth’s behavioral MR were indirectly associated with higher internalizing and externalizing behavior through higher levels of behavioral SR and (2) youth’s emotional MR were associated with higher internalizing problems through higher emotional SR, but also with lower internalizing problems through lower levels of behavioral SR. These results emphasize the importance of stimulating positive SR, by showing that they can be a protective factor for youth in residential care.

## Introduction

Studies have consistently identified that youth in residential care have more mental health problems and psychopathology than the general population. Even though only a relatively small proportion of this population presents clinically evaluated and diagnosed behavioral and mental health problems in Portugal (ISS.IP, 2020), children and youth in care are, nonetheless, a high-risk population in terms of their mental health, namely, externalizing and internalizing problems (e.g., Gearing et al., 2014; González-García et al., 2017; Magalhães and Calheiros, 2017; Campos et al., 2019). They present a relatively high prevalence of conduct disorder, attention-deficit hyperactivity disorder, depression, post-traumatic stress disorder, and generalized anxiety disorder (Heflinger et al., 2000; Tarren-Sweeney and Vetere, 2013; Rodríguez et al., 2015; Jozefiak et al., 2016). These studies also indicate that male adolescents have a higher prevalence of externalizing disorders, while female adolescents have a higher prevalence of internalizing disorders. The over-representation of males in care contributes to the predominance of externalizing disorders in this population (Schmid et al., 2008; Jozefiak et al., 2016). Regardless of gender differences, the heightened vulnerability of these youth for adjustment problems may be partially explained by pre-care (e.g., abuse and neglect; Cicchetti and Lynch, 1993; Hukkanen et al., 1999; Taussig, 2002; Richardson and Lelliott, 2003; Baams et al., 2013; Lehmann et al., 2013) and during-care experiences (e.g., placements and repeated breakdowns and staff turnover; Rutter, 2000; Attar-Schwartz, 2009; Lehmann et al., 2013).

Another factor that may partially explain these worse outcomes in this population is the social stigma associated with being in residential care (Mullan et al., 2007; Simsek et al., 2007; Villagrana et al., 2018; An et al., 2020). Social stigma has been conceptualized as a fundamental cause of health inequalities, and one of the most frequently hypothesized risk factors explaining mental health disparities among young people (e.g., Hatzenbuehler et al., 2013; Hatzenbuehler, 2016). When children and youth perceive negative stereotypes, this brings negative consequences for their wellbeing and psychological adjustment (e.g., Major and O’Brien, 2005; Pascoe and Richman, 2009; Baams et al., 2013; Puhl and King, 2013). According to Major and O’Brien’s review (2005), individuals’ negative perceptions of their social images – that is, what youth think other people in general think about them (i.e., meta-representations, MR) – and perceived stereotyping threat create involuntary stress responses that may have an effect on important outcomes, such as self-esteem, academic achievement, and mental health. However, previous studies have only examined the relationships between these two phenomena in normative populations. Previous studies analyzing the association between perceived stigma and mental health outcomes in the context of residential care have only focused on young people’s feelings of stigma (e.g., Simsek et al., 2007; An et al., 2020) and have not looked at the content of their perceptions of their social images (i.e., their perceptions of how others in general perceive them regarding specific self-relevant attributes; Calheiros et al., 2015). Thus, it remains unknown how different dimensions of MR and mental health are interrelated among adolescents in residential care. Therefore, aiming to expand existing knowledge on the risk factors for mental health problems in youth in residential care, this study intends to explore the relationship between the perceptions that youth in residential care have of their social images (MR) and their mental health.

In addition to the direct effect that youth’s MR may have on their mental health, this effect may also be mediated by youth’s self-representations (SR; i.e., the set of attributes that individuals use to describe themselves; Harter, 2015). Individuals’ perceptions of their stereotypical social images provide a fundamental input into their identity and self-development (e.g., Crocker and Quinn, 2000; Gallagher and Zahavi, 2007; Turner and Whitehead, 2008). Indeed, symbolic interactionism theorists (Cooley, 1902; Mead, 1934; Charon, 1985) have argued that the sources of self-knowledge are rooted in social interactions and experiences, and derive, in part, from how individuals perceive to be perceived by others, that is, their MR. Prior studies have shown that youth in residential care are labeled with negative social images by laypeople and professionals (e.g., Kuznetsova, 2005; Montserrat et al., 2013; Calheiros et al., 2015). Research has also revealed that others’ perceptions of youth in residential care are more negative than those of youth living in their natural home environment (Garrido et al., 2016) and that youth in residential care identify themselves as targets of negative social images (e.g., Mullan et al., 2007; Simkiss, 2013). According to the symbolic interactionism perspective (e.g., Mead, 1934), this may lead these youth to depreciate and stigmatize themselves, and to internalize others’ perceptions in their SR (Kools, 1997; Major and O’Brien, 2005; Vojak, 2009; McMurray et al., 2011). Consistent with self-stigmatization processes, defined as the internalization of negative societal attitudes about one’s social group, that have been described in the literature (e.g., Corrigan et al., 2013), a recent longitudinal study with adolescents in out-of-home care found that perceived stigmatization predicted lower self-esteem over time (An et al., 2020). However, that study also showed that, as adolescents’ perceptions of stigma decreased, their self-esteem increased. Thus, notwithstanding the hazards associated with the stigmatization of youth in residential care, such evidence points out the potential of positive social images to stimulate the development of positive self-representations in this group.

In turn, SR are associated with mental health. Studies have indicated that they can either predict better health and social behavior or function as a factor leading to internalizing (e.g., depression, suicidal tendencies, eating disorders, and anxiety) and externalizing problems (e.g., violence and substance abuse; Mann et al., 2004; Kepper et al., 2011; Silva and Calheiros, 2020). Thus, these studies suggest a possible mediation, where negative youth’s MR associate with negative SR (e.g., Calheiros et al., 2015), which, in turn, are associated with poor mental health outcomes (e.g., Mann et al., 2004; Waniel et al., 2006; Silva and Calheiros, 2020). Indeed, stigma perception has been shown to indirectly affect mental health through self-esteem (Lin et al., 2009). In the same vein, that mediation hypothesis also foresees that youth’s perceptions of their positive social images may be associated with better mental health outcomes through positive SR.

Although it has been previously assumed that SR might deteriorate due to negative processes associated with institutionalization, namely, stigmatization (Liebling, 1993; Kools, 1997; McMurray et al., 2011; An et al., 2020), other studies indicate that SR may remain stable or even become more positive (Greve and Enzmann, 2003). Fluctuations in the valence of self-representations of youth during institutionalization depend on the relation between several processes (Greve and Enzmann, 2003), such as their ability to adjust (Barendregt et al., 2015), and a set of personal and context variables (Hukkanen et al., 1999; González-García et al., 2017). Thus, in the context of residential care, youth’s age and characteristics of the residential care unit may moderate these relations. Specifically, youth’s age may play a moderating role in associations between youth’s MR and their SR. Early to middle adolescence has been associated with a higher internalization of social group norms and rules and a greater awareness of how the self is perceived by others (Harter, 2015), whereas young adults tend to exhibit self-descriptions that suggest that the self is relatively differentiated from primary social groups (e.g., Labouvie-Vief et al., 1995; McAdams, 2013). In addition, the size of the residential care unit may also moderate the path from SR to the outcome variables, since residential care settings with a lower number of youths and a lower youth/care worker ratio allow a more family-like environment, which facilitates relationship building and seems to contribute to better adjustment outcomes in youth (Lee and Thompson, 2008; Attar-Schwartz, 2009; Calheiros et al., 2020b). The size of these settings in Portugal varies significantly (GEP/MTSS, 2018; Magalhães et al., 2018; Silva et al., 2021), since there are settings that host a large number of children and smaller, more family-like, units.

**Figure 1:**
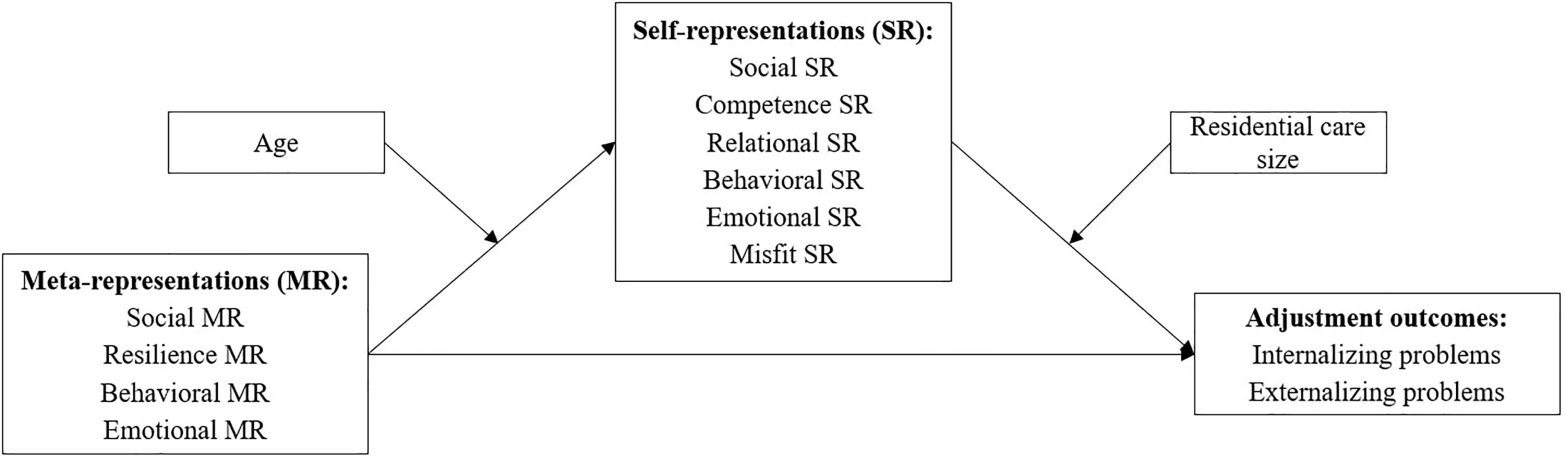
Theoretical model summarizing the predicted relationships between constructs.

The present investigation builds on a previous study focused on analyzing the associations between youth’s MR and their SR, moderated by their perceived social support from their residential care workers and from their friends (Calheiros et al., 2020a). As a complement to that study and given the lack of research regarding the link between youth’s MR and their mental health, mediated by their SR, especially with youth in residential care, this study aims to explore these associations, considering the moderating role of youth’s age and residential unit size (see Figure 1). Specifically, we hypothesized that: (1) youth’s MR are indirectly associated to mental health outcomes, through youth’s SR – whereby negative MR are associated with worse SR, which, in turn, are associated to poorer mental health outcomes; and positive MR are associated to more favorable SR, which, in turn, are associated with better mental health outcomes; (2) the indirect association between youth’s MR and mental health through SR is stronger for younger youth; and (3) the indirect associations between youth’s negative MR and poorer mental health through negative SR are stronger for youth in bigger residential care settings, while the indirect associations between youth’s positive MR and better mental health through positive SR are stronger in smaller residential care settings. Pre-care variables known to be risk factors for psychosocial problems, such as history of parental maltreatment and family socioeconomic status, will be controlled for as covariates. The number of previous placements in residential care and the length of stay in residential care will also be included in the model as covariates, given previous research showing associations between these variables and youth’s mental health outcomes. Specifically, placement change has been shown to be associated with higher levels of both internalizing and externalizing behavior problems (Aarons et al., 2010; Rosenthal and Villegas, 2010; Jones et al., 2011). As for length of stay, research has shown inconsistent findings with some studies showing a positive association with better psychosocial functioning in young people (e.g., Assouline and Attar-Schwartz, 2020), others showing no association (e.g., Heflinger et al., 2000), and others showing a negative association (e.g., Hussey and Guo, 2002). Additionally, given that prior research has shown significant sex differences in mental health outcomes of youth in residential care (Schmid et al., 2008; Jozefiak et al., 2016), youth’s sex will also be included in the hypothesized model as a covariate.

## Materials and Methods

### Research Context

In Portugal, the full implementation of a protection system focused on the family potential has not yet been established (Rodrigues et al., 2013). Thus, residential care is still the primary form of out-of-home care for children and youth in this country. The residential care system is supervised by the Ministry of Welfare and is divided into the following services: Foster Care, Generalist Residential Care Settings, and Specialized Residential Care Settings. Specialized care includes (a) Emergency Shelters, (b) Residential care to address therapeutic or educational needs (e.g., for children and youth with severe mental health problems), and (c) Autonomy apartments. Residential Care Centers are used as a long-term out of home response enforced by the child-care protection system in order to ensure the safety, wellbeing, and development of children and youth at risk (e.g., orphaned, abandoned, deprived of adequate family environment, subject to abuse, and/or neglect). To reduce placement instability, a recent change in the Portuguese law (Law n° 142/2015 of the Assembly of the Republic, 2015) determined that young people in care should only be subjected to placement change when that is in their best interest. The most recent official report characterizing the whole population of children and youth in out-of-home care in Portugal (i.e., CASA report; ISS.IP, 2020) indicates that 86% of young people in out-of-home care are living in generalist residential care settings, and about 11% are living in specialized or therapeutic residential care settings (ISS.IP, 2020). Foster care represents merely about 3% of out-of-home care. These data show the still insufficient investment in prevention and in the promotion of family foster care or therapeutic residential care as an alternative to generalist residential care (ISS.IP, 2020).

The current study was conducted in generalist residential care units. The last CASA report (ISS.IP, 2020) indicates that 72% of the young people living in these units are 12 or more years old. Overall, gender is relatively balanced (52% of males and 48% females) and the length of placement is usually high, with 34% of the children and youth living in residential care for over 4years or more. According to that report, behavioral problems have been identified in 27% of this population with particular incidence in youth aged between 15 and 17years **(**ISS.IP, 2020). Approximately 4.2% have been clinically diagnosed with a mental health problem (by a mental health professional), mostly (and similarly) among 10- to 20-year olds, although about 59% benefit from regular psychological and/or psychiatric counseling (ISS.IP, 2020). Indeed, the real rates of clinical mental health problems among this population are expected to be much higher. A recent pilot study with 59 youth in residential care in Portugal (Rodrigues et al., 2019) showed that the percentages of youth with clinical or borderline scores were 50.8% for externalizing problems (i.e., rule-breaking and aggressive behavior) and 44% for internalizing problems (i.e., depression/anxiety, depression/withdrawal, and somatic complaints) evaluated with the Youth Self-Report scale (Portuguese version, Achenbach et al., 2014). Another study with 841 children and youth in residential care in Portugal (Rodrigues, 2019), assessed with the Strengths and Difficulties Questionnaire (Goodman, 1997; Goodman et al., 2003), showed that 43.6% had psychological adjustment difficulties at the borderline and clinical levels.

### Participants and Procedures

Participants were 926 youth from 71 residential care settings aged between 12 and 25years old (*M*=16.26, *SD*=2.22). Table 1 presents a summary of the youth’s socio-demographic characteristics, namely, gender, nationality, and reason for placement in residential care. Notwithstanding the different nationalities of a small proportion of participants, all of them spoke Portuguese. Participating youth had been in the current residential care setting for 29days to 20years and 10months (*M*=3.74years, *SD*=3.71). Most (61.0%) had only been placed in the current setting, while 39% had previous out-of-home placements. Of those with previous placements, most (76.6%) had only one previous placement, 16.6% had two, and 6.6% had three or more. These data resemble the nationwide data provided in the CASA report (ISS.IP, 2020): 67% (4.700) had no previous placement experiences. Regarding psychopathology rates, 42.5% of the youth presented clinical or borderline scores for internalizing problems (15.4% borderline; 27.0% clinical), and 46.2% presented clinical or borderline scores for externalizing problems (13.0% borderline; 33.2% clinical).

**Table 1:** Socio-demographic characteristics of the study sample.

	Valid percentage
**Gender**	
Female	45.5%
Male	54.5%
**Nationality**	
Portuguese	92.4%
African countries	
Guineans	2.7%
Cape Verdean	2.0%
Angolan	0.8%
San Tomean	0.2%
Moroccan	0.1%
**European countries**	
German	0.3%
Ukrainian	0.3%
Romanian	0.2%
Spanish	0.1%
**Central/South American countries**	
Brazilian	0.5%
Guatemalan	0.2%
**Reason for placement in residential care**	
Neglect	49.5%
Exposure to harmful behaviors	45.2%
Deviant behaviors	27.2%
Psychological abuse	16.7%
Physical Abuse	15.3%
Abandonment	10.5%
Sexual abuse	4.6%

The residential units hosted between three and 53 youth (*M*=18.05, *SD*=10.44). Regarding the staff, these units had between one and four case managers (e.g., social workers and psychologists) and between one and 15 care workers. The mean ratio was between one and 41 youth per care worker. These were mainly long-term residential care units (60.6%) from urban areas (67.6%).

Following approval from the Ethics Commission of the University, as part of a broader research project focused on youth’s SR, this study was developed in 71 generalist residential care units, representing 17 of the 18 districts of Portugal (94.4%). Formal contacts allowed the necessary authorizations to collect the data, and all youth placed in these units for more than 1 month, aged 12 or more years old, were invited to participate, except if they presented major cognitive difficulties (information given by the residential unit director). Consent for youth’s participation was first obtained from their respective residential unit director, who is the person responsible for accompanying and adjudicating youth’s formal decisions in the context of residential care. All youths who met the inclusion criteria and were authorized to participate by their residential unit director were included in the study, except those who declined to participate. Overall, youth’s consent and participation ranged from 13.3 to 100% (*M*=68.84, *SD*=24.11) across residential care settings. Data collection with youth was conducted in groups of 3–20 participants (a mean of 10 youth per group and a ratio of at least one researcher to 10 youth). The goals of the study and instructions for filling out the instruments for data collection were explained at the beginning of the data collection session, and the researcher was always present to answer any questions and provide youth with any help or assistance whenever necessary. Information regarding anonymity and confidentiality was also given at the beginning of the session, and youth signed an informed consent form prior to their participation.

Youth with any reading and comprehension difficulties were previously identified by their residential care workers and were individually interviewed by one of the researchers, following the data collection protocol (195 individual interviews conducted, 21.1%). At the end of each data collection session, youth put their questionnaires, which were completed with the research team, in a closed box, in order to assure them that their answers would not be viewed by the residential care unit professionals. The questionnaires filled out by the residential care workers, the case managers, and the directors were collected on the same day of the youth data collection. They had also been previously informed regarding the aims of the research, anonymity and confidentiality of the data, and signed an informed consent form prior to their participation. Data were collected between 2015 and 2016.

### Instruments

#### Self-Representations

To measure youth SR, we used the Self-Representations Questionnaire for Youth in Residential Care (SRQYRC; Patrício et al., 2016). The questionnaire is composed of 23 items, organized in six dimensions (Social – nice, friend, helpful, and funny; Competence – intelligent, hard-working, committed, and competent; Relational – cherished, protected, and loved; Behavioral – aggressive, recalcitrant, misbehaved, conflicting, problematic, and stubborn; Emotional – depressed, traumatized, sad, and lonely; and Misfit – misfit and neglected) measuring youth’s SR on positive social, competence and relational attributes, and on negative behavioral, emotional, and misfit attributes. Participating youth were asked to rate each attribute on a 5-point scale, indicating how descriptive it was of themselves (1=I am definitely not like that; 5=I am totally like that). This measure was tested in a previous study and showed good psychometric properties, namely, adequate model fit (*χ*^2^/*df*=2.031, CFI=0.927, TLI=0.916, RMSEA=0.050), reliability (except on misfit dimension; social *α*=0.81, competence *α*=0.75, relational *α*=0.72, behavioral *α*=0.80, emotional *α*=0.75, and misfit *α*=0.55), mean inter-item correlations (social 0.52, competence 0.43, relational 0.47, behavioral 0.40, emotional 0.43, and misfit 0.38), and construct validity (Patrício et al., 2016). In this sample, reliability evidence was similar to that obtained previously by the original scale authors, varying between 0.55 and 0.81.

#### Youth’s Meta-Representations

Following the classic paradigm to assess individuals’ representations regarding others’ representations of them (e.g., Nurra and Pansu, 2009), the questionnaire used to measure youth’s MR was adapted from the SRQYRC (Patrício et al., 2016): Instead of rating themselves regarding each attribute, youth were asked to rate how descriptive each attribute was of the way people in general think about them, on a 5-point scale, (1=People in general think I am definitely not like that; 5=People in general think I am totally like that). An exploratory factor analysis of this measure resulted in a final structure of 19 attributes, organized in four dimensions (Social – nice, friend, and helpful; Resilience – courageous, fighter, and protected; Behavioral – recalcitrant, stubborn, misbehaved, aggressive, conflicting, and angry; and Emotional – depressed, lonely, traumatized, sad, neglected, low self-esteem, and abandoned) measuring youth’s MR (i.e., to which extent youth think that people in general perceive them as sociable and resilient, or as having behavioral and emotional problems). Since this measure was adapted to this study, we tested its structure within this study’s sample. This scale’s structure was tested with a Confirmatory Factor Analysis, which showed an adequate model fit (*χ*^2^/*df*=2.169, CFI=0.936, TLI=0.924, RMSEA=0.059), and the four dimensions showed good reliability (Social *α*=0.87, Resilience *α*=0.70, Behavioral *α*=0.84, and Emotional *α*=0.83).

#### Internalizing and Externalizing Problems

To measure youth’s mental health, the Child Behavior Checklist (Portuguese version, Achenbach et al., 2014) was filled in by the residential youth care workers who spent the most amount of time each day with each youth. In the cases, where there was more than one care worker spending the same amount of time with the youth, a number was attributed to each care worker, and then, one of them was randomly selected with the RANDBETWEEN function in excel. The 118 items of this measure were used, which are rated as not true (0), somewhat or sometimes true (1), or very true or often true (2). Although this measure allows for the evaluation of various mental health dimensions, our analysis will focus only on the Internalizing (Anxious/depressed, Withdrawn/depressed, and Somatic complaints) and Externalizing scales (Rule-breaking behavior and Aggressive behavior). This measure has been tested in previous studies and has shown adequate psychometric properties, namely, adequate reliability and good model fit indexes (CFI=0.919, TLI=0.917, RMSEA=0.020; Achenbach, 1991; Achenbach and Rescorla, 2001; Achenbach et al., 2014).

#### Individual Characteristics and Size of the Residential Care Unit

The professionals responsible for the case management of each youth filled in a questionnaire asking for socio-demographic data, such as youth’s gender, birthday date, and placement date. To measure the size of the residential care unit, the respective director filled out a questionnaire asking for indicators such as number of youths currently placed in this unit and ratio of youth per residential care worker.

#### Previous Maltreatment

To measure pre-care parental maltreatment, the case manager of each youth completed the Child Maltreatment Questionnaire (Calheiros et al., 2021). This questionnaire is composed of 19 items organized in four dimensions: Neglect – lack of physical provision, Physical and psychological abuse, Emotional and educational maltreatment, and Neglect – lack of supervision. This measure has been tested in previous studies and has shown good psychometric properties, namely, adequate model fit (*χ*^2^/*df*=3.52, CFI=0.905, GFI=0.905, TLI=0.886, RMSEA=0.072) and reliability (Neglect – Lack of provision *α*=0.76, Neglect – Lack of supervision *α*=0.84, Emotional and Educational Maltreatment *α*=0.76, and Physical and Psychological abuse *α*=0.81; Calheiros et al., 2021). In the final part of the questionnaire, based on item evaluation and on the information available in the youth’s record, the professionals were asked to evaluate on a yes/no scale if the youth was subjected to neglect, physical or psychological abuse, or sexual abuse. These last items were computed in a variable ranging from 0 (no abuse registered) to 3 (all forms of abuse registered), which was used as a control variable.

#### Socioeconomic Status

To measure pre-care socioeconomic family status, the professional staff responsible for the case management of each youth reported on variables such as monthly income, income source, habitation, residence place, and parental academic level (Appendix 1). A Multiple Correspondence Analysis (MCA) was conducted to transform the categorical variables in order to compute a composite variable. After optimal quantification, the variables on the first dimension (axis) showed adequate reliability (*α*=0.74). From these category quantifications, a factorial score was calculated for each participant and therefore, a new composite variable was computed to measure socioeconomic status, and the object scores were saved as a new (quantitative) variable which was used as a control variable in the following analyses.

### Data Analysis

First, a MCA was performed to compute a composite variable of socioeconomic family status. MCA is a multivariate method that assesses the relational structure between input variables (Gifi, 1996; Greenacre, 2007; Carvalho, 2008). MCA is similar to Principal Component Analysis, but it is applied to categorical variables. MCA transforms categorical input variables using an optimal scaling procedure and assigns an optimal quantification to each category of each one of the input variables. Using the optimal quantifications, a factorial score is calculated for each object, which includes all of the categories that define its profile. Therefore, a new composite and quantitative variable are obtained.

The following analyses included descriptive statistics and bivariate correlations of the predictor, criterion, mediator, and moderator variables. Then, to examine whether SR mediate the relation between youth’s MR and youth mental health, two multiple mediation analyses were conducted, one for each criterion variable – internalizing problems and externalizing problems. Finally, moderated mediation analyses were conducted to test the moderating role of age and residential care unit size. In all analyses, we controlled for youth’s sex, previous family socioeconomic status, number of placement changes, length of placement, and previous maltreatment experiences, by including them as covariates in the models.

These analyses were conducted using a non-parametric method (*bootstrap*) based on recommendations by Preacher et al. (2007) and Hayes (2015) through PROCESS macro for SPSS version 20. We generated 5,000 bootstrap samples to yield a 95% bias-corrected confidence interval (CI) of the indirect effect and of the conditional indirect effect. If the CI for the indirect effect obtained by *bootstrap* estimation does not include zero, the effect is significant, and the indirect associations is established. Similarly, if the CI for the effect of moderated mediation does not include zero, the moderated indirect associations are established.

## Results

### Descriptive Statistics and Bivariate Correlations

Descriptive statistics (*M*, *SD*) and bivariate correlations are presented in Table 2. Globally, positive dimensions of youth’s MR (Social and Resilience) were positively related to positive SR dimensions (Social, Competence, and Relational) and negatively related to negative SR dimensions (Behavioral, Emotional, and Misfit), while negative dimensions of youth’s MR (Behavioral and Emotional) were negatively related to positive SR dimensions and positively related to negative SR dimensions. Regarding the criterion variables, positive dimensions of youth’s MR and positive SR dimensions were negatively related to internalizing problems, while negative youth’s MR and SR dimensions were positively related to internalizing and externalizing problems. Externalizing problems were positively related to internalizing problems. As for the moderators, age was positively related to competence SR and negatively related to externalizing problems; youth/care worker ratio was positively related to the resilience dimension of youth’s MR and negatively related to internalizing problems; and number of youths living in the residential care unit was negatively related to emotional SR and internalizing problems. Considering the number of significant correlations with the number of youths living in the residential care unit, and its positive correlation with the youth/care worker ratio, we decided to keep only the number of youths living in the residential care unit as a moderator in the subsequent analysis.

**Table 2:** Descriptive statistics (M, SD) and bivariate correlations.

	1	2	3	4	5	6	7	8	9	10	11	12	13	14	15
1.MRSoc															
2.MRRes	0.504^**^														
3.MRBeh	−0.176^**^	−0.029													
4.MREmo	−0.312^**^	−0.229^**^	0.437^**^												
5.SRSoc	0.531^**^	0.469^**^	−0.100^**^	−0.239^**^											
6.SRCom	0.356^**^	0.433^**^	−0.118^**^	−0.119^**^	0.476^**^										
7.SRRel	0.400^**^	0.496^**^	−0.080^*^	−0.189^**^	0.468^**^	0.383^**^									
8.SRBeh	−0.129^**^	0.000	0.711^**^	0.258^**^	−0.100^**^	−0.168^**^	−0.016								
9.SREmo	−0.127^**^	−0.093^*^	0.246^**^	0.515^**^	−0.166^**^	−0.078^*^	−0.151^**^	0.318^**^							
10.SRMis	−0.265^**^	−0.145^**^	0.162^**^	0.388^**^	−0.203^**^	−0.101^**^	−0.194^**^	0.222^**^	0.353^**^						
11.Intern	−0.104^**^	−0.152^**^	0.033	0.243^**^	−0.142^**^	−0.094^*^	−0.145^**^	0.130^**^	0.300^**^	0.108^**^					
12.Extern	−0.059	0.033	0.284^**^	0.097^*^	−0.072	−0.070^**^	−0.001	0.374^**^	0.083^*^	0.104^**^	0.322^**^				
13.Age	0.007	−0.012	−0.065	0.038	0.029	0.201^**^	−0.018	−0.068	0.063	0.023^**^	0.066	−0.125^**^			
14.Ratio	0.030	0.088^*^	−0.020	−0.048	−0.004	0.058	0.051	−0.049	−0.034	0.024^**^	−0.100^*^	−0.012	−0.058^**^		
15.NYRC	0.036	−0.026	−0.015	−0.067	0.069	0.041	0.029	−0.028	−0.108^**^	0.021^**^	−0.130^**^	−0.020	−0.082^**^	0.405^**^	
*M*	4.12	3.83	2.45	1.82	4.17	3.68	3.84	2.53	2.25	1.78	10.58	14.57	16.26	4.48	24.09
*SD*	0.77	0.82	0.89	0.70	0.64	0.72	0.84	0.79	0.84	0.82	7.33	11.76	2.22	7.74	12.66

MR Soc, Social youths’ awareness of others’ representations about them; MR Res, Resilience youths’ awareness of others’ representations about them; MR Beh, Behavioral youths’ awareness of others’ representations about them; MR Emo, Emotional youths’ awareness of others’ representations about them; SRSoc, Social Self-Representations; SRCom, Competence Self-Representations; SRRel, Relational Self-Representations; SRBeh, Behavioral Self-Representations; SREmo, Emotional Self-Representations; SRMis, Misfit Self-Representations; Intern, Internalizing problems; Extern, Externalizing problems; NYRC, Number of youths living in the residential care unit. ^*^p<0.05 and ^**^p<0.01.

### Multiple Mediation Analyses

Regarding internalizing problems (Table 3), mediation analyses revealed significant indirect effects of the youth’s emotional MR on internalizing problems through emotional and behavioral SR; and of youth’s behavioral MR on internalizing problems through behavioral SR. Specifically, youth that think that others in general perceive them as having more behavioral problems also reported higher levels of negative behavioral SR (Behavioral MR → Behavioral SR *B*=0.670, *SE*=0.028, *p*<0.001, 95% CI=0.615, 0.724) and youth with higher behavioral SR revealed more internalizing problems (Behavioral SR → Internalizing *B*=1.528, *SE*=0.581, *p*=0.009, 95% CI=0.387, 2.670). Thus, although the direct effect of the Behavioral dimension of youth’s MR on internalizing problems is negative (i.e., youth that think that others in general perceive them as having more behavioral problems have fewer internalizing problems), when the Behavioral SR is added as a mediator, the indirect effect of the Behavioral dimension of youth’s MR on internalizing problems through Behavioral SR is positive. Since the behavioral and emotional dimensions of youth MR had a significant direct effect on internalizing problems, emotional and behavioral SR partially mediated these relations.

**Table 3:** Multiple mediation models.

Covariates, predictors, and mediators	Dependent variables			
	Internalizing problems		Externalizing problems	
	Coeff. (SE)	95% CI	Coeff. (SE)	95% CI
**Covariates**				
Sex^1^	−1.712 (0.611)^**^	[−2.888, −0.514]	2.152 (0.903)^*^	[0.379, 3.927]
Previous maltreatment experiences	−0.040 (0.415)	[−0.854, 0.774]	−0.312 (0.632)	[−1.554, 0.931]
SES	−0.098 (0.292)	[−0.671, 0.475]	−0.061 (436)	[−0.917, 0.795]
Placement length	0.049 (0.078)	[−0.104, 0.203]	−0.171 (0.114)	[0.395, 0.054]
Number of previous placements	1.022 (439)^*^	[0.160, 1.884]	−0.012 (0.674)	[−1.312, 1.336]
**Predictor: Social MR**				
Total effect	−0.252 (0.443)	[−1.181, 0.677]	−0.571 (0.780)	[−1.965, 0.823]
Direct effect	−0.058 (0.525)	[−1.088, 0.973]	−0.148 (0.776)	[−1.377, 1.672]
**Indirect effect**				
Social SR	−0.327 (0.305)	[−0.946, 0.236]	−0.180 (0.409)	[−0.923, 0.448]
Competence SR	0.098 (0.122)	[−0.127, 0.350]	−0.078 (0.351)	[−0.375, 0.188]
Relational SR	−0.088 (0.137)	[−0.388, 0.157]	−0.053 (0.181)	[−0.431, 0.286]
Behavioral SR	−0.089 (0.091)	[−0.321, 0.022]	−0.332 (0.219)	[−0.833, 0.050]
Emotional SR	0.125 (0.095)	[−0.035, 0.342]	−0.027 (0.064)	[−0.173, 0.097]
Misfit SR	0.086 (0.072)	[−0.045, 0.244]	−0.049 (0.112)	[−0.304, 0.151]
**Predictor: Resilience MR**				
Total effect	−0.993 (0.408)^*^	[−1.794, −0.191]	0.378 (0.691)	[−0.839, 1.596]
Direct effect	−0.822 (0.453)	[−1.711, 0.068]	0.482 (0.691)	[−0.875, 1.839]
**Indirect effect**				
Social SR	−0.156 (0.144)	[−0.454, 0.114]	−0.097 (0.188)	[−0.494,0.246]
Competence SR	0.136 (0.164)	[−0.186, 0.461]	−0.124 (0.218)	[−0.584,0.278]
Relational SR	−0.128 (0.184)	[−0.498, 0.242]	−0.078 (0.250)	[−0.543,0.436]
Behavioral SR	0.028 (0.057)	[−0.078, 0.155]	0.177 (0.173)	[−0.140, 0.543]
Emotional SR	−0.078 (0.155)	[−0.190, 0.094]	0.013 (0.039)	[−0.055, 0.109]
Misfit SR	−0.016 (0.029)	[−0.091, 0.025]	0.006 (0.033)	[−0.055, 0.084]
**Predictor: Emotional MR**				
Total effect	2.890 (0.491)^***^	[1.926, 3.854]	−0.481 (0.748)	[−1.951, 0.989]
Direct effect	2.002 (0.579)^***^	[0.865, 3.149]	−0.038 (0.882)	[−1.769, 1.693]
**Indirect effect**				
Social SR	0.057 (0.067)	[−0.057, 0.211]	0.024 (0.061)	[−0.080, 0.171]
Competence SR	0.032 (0.049)	[−0.053, 0.144]	−0.042 (0.085)	[−0.241, 0.113]
Relational SR	0.009 (0.033)	[−0.057, 0.086]	0.012 (0.053)	[−0.094, 0.129]
Behavioral SR	−0.114 (0.088)	[−0.324, −0.014]	−0.327 (0.200)	[−0.752, 0.053]
Emotional SR	1.080 (0.327)	[0.474, 1.754]	−0.238 (0.450)	[−1.130, 0.634]
Misfit SR	−0.176 (0.140)	[−0.464, 0.095]	0.129 (0.289)	[−0.415, 0.729]
**Predictor: Behavioral MR**				
Total effect	−0.828 (0.371)^*^	[−1.556, −0.100]	3.675 (0.575)^***^	[2.546, 4.805]
Direct effect	−1.791 (0.530)^***^	[−2.832, −0.751]	0.484 (0.796)	[−1.080, 2.048]
Indirect effect				
Social SR	−0.001 (0.034)	[−0.095, 0.052]	−0.001 (0.031)	[−0.067, 0.066]
Competence SR	−0.045 (0.060)	[−0.182, 0.065]	0.046 (0.085)	[−0.111, 0.228]
Relational SR	0.000 (0.024)	[−0.051, 0.050]	−0.005 (0.031)	[−0.085, 0.048]
Behavioral SR	1.024 (0.516)	[0.009, 2.027]	3.151 (0.606)	[1.955, 4.332]
Emotional SR	−0.004 (0.064)	[−0.137, 0.123]	0.004 (0.029)	[−0.064, 0.068]
Misfit SR	−0.002 (0.025)	[−0.062, 0.047]	−0.005 (0.033)	[−0.093, 0.046]
Total effect model *R*^2^a=	0.129		0.096	
Direct effect model *R*^2^a=	0.176		0.152	

1 Sex: 1 – Male and 0 – Female. ^*^p<0.05, ^**^p<0.01, and ^***^p<0.001.

Similarly, youth that think that others perceive them as having more emotional problems reported higher levels of negative emotional SR (Emotional MR → Emotional SR *B*=0.633, *SE*=0.047, *p*<0.001, 95% CI=0.598, 0.725) and youth with higher emotional SR displayed more internalizing problems (Emotional SR → Internalizing *B*=1.705, *SE*=0.459, *p*<0.001, 95% CI=0.804, 2.607). Thus, the indirect effect of the emotional dimension of youth’s MR on internalizing problems through emotional SR is positive.

Results also revealed a significant total effect of youth’s resilience MR on internalizing problems. That is, youth that think that others in general perceive them as having more resilience skills display fewer internalizing problems.

**Figure 2:**
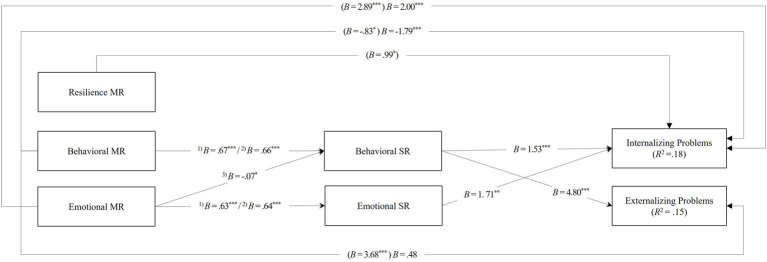
Model examining the associations between youth’s meta-representations (MR) and their mental health (i.e., internalizing and externalizing problems) mediated by their self-representations (SR). Coefficients in brackets refer to the total effects of MR domains on internalizing and externalizing problems. ^1)^Estimate obtained in the model for internalizing problems. ^2)^Estimate obtained in the model for externalizing problems. ^3)^Estimate obtained in both models. ^*^p<0.05, ^**^p<0.01, and ^***^p<0.001.

Regarding externalizing problems (Table 3), mediation analyses revealed a significant indirect effect of the behavioral dimension of youth’s MR on externalizing problems through behavioral SR. Since the direct effect was non-significant, the behavioral SR totally mediated this relation. That is, youth that think that others in general perceive them as having more behavioral problems also reported higher levels of negative behavioral SR (Behavioral MR → Behavioral SR *B*=0.656, *SE*=0.029, *p*<0.001, 95% CI=0.599, 0.713) and youth with higher behavioral SR displayed more externalizing problems (Behavioral SR → Externalizing *B*=4.801, *SE*=0.866, *p*<0.001, 95% CI=3.100, 6.503). Figure 2 depicts the significant total, direct, and indirect effects.

As for the covariates, results showed that being female is associated with higher levels of internalizing problems, while being male is associated with higher levels of externalizing problems. Finally, higher placement change was associated with higher levels of internalizing problems (Table 3).

### Moderated Mediation Analyses

Based on the results of the multiple mediation analyses, the statistically significant indirect effects obtained were then tested for moderated mediation. Youth’s age was analyzed as a first stage moderation (path a), and the size of the residential care unit as a second stage moderation (path b). Mean centering was used for product terms. Moderator’s values of low and high are the mean plus/minus one standard deviation. As can be seen in Table 4, no moderated mediation effects were found. According to the index of moderated mediation, neither age nor the number of youths living in the residential care unit significantly moderated the indirect effect of the dimensions of youth’s MR on youth internalizing and externalizing problems through youth’s SR dimensions.

**Table 4:** Moderated mediation models.

Indirect effect	Index of moderated mediation			
	Age		NYRC	
	Coeff. (SE)	95% CI	Coeff. (SE)	95% CI
Emotional MR → Emotional SR → Internalizing	−0.063 (0.042)	(−0.152, 0.013)	0.028 (0.020)	(−0.014, 0.067)
Behavioral MR → Behavioral SR → Internalizing	−0.001 (0.021)	(−0.044, 0.045)	−0.019 (0.020)	(−0.060, 0.019)
Behavioral MR → Behavioral SR → Externalizing	−0.055 (0.034)	(−0.118, 0.015)	−0.055 (0.034)	(−0.118, 0.015)

*NYRC, Number of youths living in the residential care unit*.

## Discussion

In this study, we aimed to explore the associations between the MR of youth in residential care and their mental health, considering the mediating role of SR domains in those associations. Additionally, we aimed to test if these relations were moderated by youth’s age and residential care size. Although we have not found support for the second and third hypotheses (i.e., the moderated mediation pathways), our findings supported our first hypothesis by revealing the mediating role of several domains of youth’s SR in those associations.

Specifically, we found that youth that think that others in general perceive them as having more behavioral problems reveal more externalizing and internalizing problems and that this association was mediated by higher levels of negative behavioral SR. Likewise, youth that think that others in general perceive them as having more emotional problems, also perceive themselves as having more emotional problems, and in turn have more internalizing problems. Taken together, these findings add to the empirical evidence on the risk factors associated with youth’s mental health in residential care by demonstrating that youth’s perceptions of their social images (i.e., MR) are associated with their internalizing and externalizing problems. Findings also complement a previous study (Calheiros et al., 2020a) analyzing the link between youth’s MR and SR moderated by social support, by showing that youth’s SR not only are related to their MR but also function as mediators of associations between youth’s MR and their mental health.

Findings also revealed that the dimensions of youth’s MR and SR with stronger significant associations with their mental health outcomes are semantically related to the mental health dimension to which they are associated. Indeed, youth’s behavioral MR showed a stronger association with externalizing problems, while youth’s emotional MR showed a stronger association with internalizing problems. Moreover, emotional SR were the main mediator for internalizing problems and behavioral SR were the main mediator for externalizing problems. These results are consistent with studies with other types of samples demonstrating that a specific SR domain was more strongly associated with outcomes that are relevant to that domain (Marsh and Craven, 2006). Namely, prior research has shown that specific behaviors are best predicted by specific self-esteem domains connected to those behaviors (Rosenberg et al., 1995). In line with that study, a meta-analysis on the effectiveness of self-concept interventions demonstrated that specific self-concept domains more logically related to the intended aims of the intervention had larger effects than those less logically related or global self-esteem (O’Mara et al., 2006). Consistent with this body of research, findings of this study support the domain-specific nature of associations among MR, SR, and behaviors.

Regarding the covariates, the results showing that females reveal higher levels of internalizing problems while males reveal higher levels of externalizing problems are consistent with previous research documenting sex differences in mental health outcomes of youth in residential care (Schmid et al., 2008; Jozefiak et al., 2016). Similarly, the positive association between the number of previous placements and youth’s internalizing problems is also in line with existing evidence on the detrimental role of placement change on the mental health of young people in out-of-home care (Aarons et al., 2010; Rosenthal and Villegas, 2010; Jones et al., 2011).

The significant direct associations between MR and mental health found in this study also merit mention. Specifically, while the indirect effect of youth’s behavioral MR on internalizing problems (described above) was positive, youth’s behavioral MR also showed a negative direct association with those problems, indicating that youth that think that others in general perceive them as having more behavioral problems display lower levels of internalizing problems. The opposite directions of the direct and indirect effect of youth’s behavioral MR on their internalizing problems indicate a competitive mediation (Zhao et al., 2010) and point to other possible mediators that might explain that negative association. For instance, based on the identity threat model of stigma (Major and O’Brien, 2005), it could be that youth’s perceptions of being perceived as more behaviorally problematic, being identity threatening (i.e., perceived as harmful for their social identity), might instigate the use of active coping responses (e.g., seeking for the support of residential care worker) that may protect them from developing internalizing problems. Additionally, youth’s emotional MR were also directly and positively associated with those problems. That is, youth that think that others in general perceive them as having more emotional problems also displayed more emotional problems, regardless of the mediating role of their emotional SR. Thus, in addition to this mediator, other emotional, cognitive, and or behavioral responses might be underlying that association. Finally, results also revealed that youth that think that others in general perceive them as having more resilience skills have fewer internalizing problems. It might be that youth’s perceptions of being seen as resilient might stimulate their confidence in overcoming their challenging circumstances, thus promoting their positive adaptation and mental health. Future studies should explore these alternative hypotheses of possible mechanisms that might account for these direct effects.

Notwithstanding the need for future work, this study reinforces the idea that members of stigmatized groups who are aware of negative social images may incorporate those images into their SR (Crocker and Major, 1989) and that when negative stereotypes are perceived by children and youth, they may have negative consequences for their wellbeing and psychological adjustment (e.g., Major and O’Brien, 2005; Baams et al., 2013; Puhl and King, 2013). This study also suggests that this association is partially explained by the internalization of others’ reflected appraisals on SR. Thus, in line with the internalization perspectives (Kools, 1997), we hypothesize that youth in care might perceive and internalize the negative social images associated with them. The internalization of these images as a negative self-concept might contribute to explain the higher levels of mental health problems of youth in residential care when compared to youth in normative contexts, consistently documented in the literature of this field (Hukkanen et al., 1999; Heflinger et al., 2000; Tarren-Sweeney and Vetere, 2013; Rodríguez et al., 2015; Jozefiak et al., 2016).

By the same token, these findings also suggest that youth’s internalizations of positive meta-perceptions in their SR are associated with better mental health outcomes. Actually, altogether, the pattern of associations obtained in this study clearly suggests that the more positive youths’ perceptions of their social images are in each of the domains evaluated, the more positive their SR, and the better their mental health outcomes. As such, findings of this study bear important practical implications for interventions both at the community level and in residential care settings. Specifically, these results emphasize the importance of raising awareness, among the overall community and residential care professionals, about the existence of social images of youth in residential care and their potential effect on these youth regarding both the way they perceive themselves and their mental health outcomes. These results highlight the need for a shift of the discourse about the residential care environment toward a more positive perspective, focused on its strengths and potential, so that in can begin to be seen as a more positive environment for the development of the young people in care (Arpini, 2003). Residential care leavers are one of the most socially excluded groups of young people in society. The stigma associated with a residential care history is one of the main predictors of exclusion (Ibrahim and Howe, 2011): Negative social images can negatively impact the reintegration of residential care leavers not only *via* social discrimination, but also through self-imposed isolation and limitation of social exposure to avoid discriminatory or stigmatizing situations. Thus, at the community level, there is a need to deconstruct the negative social images of youth in residential care and increase the social awareness of the negative consequences they may produce, namely, at the level of their reintegration in their communities. To that end, efforts should be made to provide young people with environments where their skills and strengths can be expressed and acknowledged (Noble-Carr and Woodman, 2018).

Interventions aimed at promoting youth’s positive sense of self should also include the residential care staff as main agents. These professionals must be mobilized to avoid negatively labeling and stereotyping these young people, since social stereotypes of young people in care cannot be reframed as long as residential care professionals reinforce such beliefs (Hodas, 2005). Thus, professionals training programs should help residential care staff recognize their biases, raise their awareness on youth’s normative development processes, and stimulate the development of adequate strategies for dealing with the challenges posed by this development phase. Residential youth care workers should be especially targeted in these interventions, since they are the adult figures who are in daily contact with the youth in care, and one of the main and closest support providers in their lives (Bastiaanssen et al., 2014; Lanctôt et al., 2016; Sulimani-Aidan, 2016; Silva et al., 2021). Thus, residential care workers should focus more on the positive aspects and abilities of the young people in care and prepare them to cope with their perceived negative social images, by helping them make realistic appreciations of their strengths and weaknesses (Harter, 2015). To that end, in their interactions youth the youth in care, residential care workers should be trained to communicate approval contingent on youth’s adequate behavior so as to stimulate accurate perceptions of their positive attributes contingent on palpable achievements. This recognition of competence would help youth own these new positive identity inputs, thereby enabling the development of a secure and realistic positive identity (Hiles et al., 2014; Smith et al., 2017; Noble-Carr and Woodman, 2018; Marshall et al., 2020). Not less importantly, care workers should also be trained in providing youth with constructive feedback regarding their negative attributes, so as to continually motivate them for self-improvement.

For these strategies to be effective, they must be implemented within genuine caring relationships with the youth in care (Smith et al., 2017; Marshall et al., 2020). Therefore, efforts should be made to promote relationship-based practice in residential care (Ruch et al., 2010; Cahill et al., 2016), by training residential care workers in building positive developmental relationships with these young people (Holden and Sellers, 2019). Such relationships are paramount to create an effective therapeutic milieu in residential care settings that can scaffold youth’s potential and actively support the development of a positive sense of self (Holden, 2009; Whittaker et al., 2016; Holden and Sellers, 2019; Izzo et al., 2020). Considering that in Portugal, generalist residential care still accounts for the majority of out-of-home care placements, a shift in public policy is needed toward promoting the integration of therapeutic residential care models, following the guidelines provided by Whittaker et al. (2016) and by specific evidence-based programs, such as the CARE model (Holden, 2009; Holden et al., 2014).

An important limitation of this study is that the study design does not allow inferences to be made about the causality of these effects. Since this is a cross-sectional study, we cannot conclude about causality. Therefore, while youth’s self-perceptions may, indeed, be explained by their meta-perceptions, they may also predict their meta-perceptions (Kenny et al., 1994). Additionally, although youth’s SR may precede their internalizing and externalizing behaviors, they may also be affected by them. Indeed, self-fulfilling prophecies may be at stake here, whereby the targets of the reflected appraisals come to behave in ways that are consistent with the expectations of others and may alter their self-concepts as result of this behavior (Crocker and Major, 1989). Thus, future studies analyzing the direction of effects hypothesized in this study should employ longitudinal designs, considering the potential role of youth’s baseline mental health, so as to provide a more robust empirical test of that causal order. Future studies should also control the generalized other about whom young people were thinking when they completed the questionnaire. Youth were asked to think about how people in general think about them, but some may have thought about family and friends, others about people in the community, and others about people at school, among others. It would also be important to compare the role of general others’ reflected appraisals with that of specific others’ reflected appraisals, given that prior studies have indicated that certain people have a greater influence on certain SR dimensions than others (Cole, 1991; Branje et al., 2003; Bois et al., 2005; Pfeifer et al., 2009). Finally, this study did not consider potential peer contagion processes within the residential care settings, which have been shown to be relevant in understanding externalizing problems in residential care youth (Dishion et al., 1999, Ryan et al., 2008). Thus, future studies focused on analyzing the pathways hypothesized in this study should also consider potential peer contagion effects on youth’s externalizing problem behavior. It would also be important in future studies to calculate the effect of setting on the hypothesized model, through a multilevel analysis. To that end, researchers should make efforts to ensure the participation of at least 30 youth per residential setting (i.e., level-2 unit) so as to achieve sufficient power to detect cross-level interactions (Shen, 2016).

## Conclusion

In sum, findings of this study showed that the perceptions that youth in residential care have of their social images (i.e., MR) are related to their SR and that specific SR domains are related to youth’s mental health outcomes. Specifically, perceiving themselves as neither behaviorally nor emotionally problematic is associated with less internalizing and externalizing problems. Considering these results, this study highlights the importance of stimulating positive SR in youth in residential care, given their positive association with their mental health. Among other strategies suggested by Harter (1999), and based on this study’s results, we underline that it is important to encourage the belief that positive SR can be achieved, increase awareness of the origins of negative self-perceptions, and promote the internalization, by the youth in care, of others’ positive opinions of them.

## Data Availability Statement

The data that support the findings of this study are not available because of strict ethical restrictions, due to the study sample being from a vulnerable population. Requests to access the datasets should be directed to maria.calheiros@psicologia.ulisboa.pt.

## Ethics Statement

This project was approved by the Ethics Commission of Lisbon University Institute (ISCTE-IUL), Process no 2014-7. This study was approved by the Ethics Commission of Faculdade de Psicologia, Universidade de Lisboa (Protocol 6/2020). The procedures used in this study adhere to the tenets of the Declaration of Helsinki. Written informed consent to participate in this study was provided by the participants’ legal guardian/next of kin.

## Author Contributions

MC designed the study and contributed to the conceptualization, data collection, manuscript writing, and edition of the final manuscript. CS provided crucial additional inputs on the conceptualization, research context description, manuscript writing, and edited the final manuscript. JP contributed to the study conceptualization, data collection, analysis, and manuscript writing. HC contributed to data analysis and results writing. All authors contributed to the article and approved the submitted version.

## Funding

This work was funded by the Portuguese National Science Foundation (FCT - PTDC/MHC-PSC/4122/2012), and co-funded by the Lisbon 2020 Program, Portugal 2020 and the European Union, through the European Regional Development Fund (ERDF) and the State Budget through Fundação para a Ciência e Tecnologia (PTDC/CED-EDG/30373/2017).

## Conflict of Interest

The authors declare that the research was conducted in the absence of any commercial or financial relationships that could be construed as a potential conflict of interest.

## Publisher’s Note

All claims expressed in this article are solely those of the authors and do not necessarily represent those of their affiliated organizations, or those of the publisher, the editors and the reviewers. Any product that may be evaluated in this article, or claim that may be made by its manufacturer, is not guaranteed or endorsed by the publisher.

## Appendix 1 Socioeconomic Status Items And Categories

**Table:**

Item	Categories
Mensal income	1=<500€; 2=500–1000€; 3 = >1000€
Income source	1=irregular income, from allowance; 2=weekly income; 3=mensal income, heritage, fortune acquired, independent worker income
Habitation	1=house without the necessary living conditions, “shack” with inappropriate living conditions; 2=house with the necessary living conditions; 3=comfortable house
Residence place	1=Neighborhood of low value, close to factories/ports/contaminated water/tents; Neighborhood with “shack, in an unhealthy suburban area, with very little value; 2=Neighborhood with old buildings, less valued and less comfortable than the next; 3=Residential neighborhood with well-preserved houses, large, wooded avenues, moderate value zone
Parental academic level	1=Elementary school; 2=Middle school; 3=High school or Higher education

## References

[ref1] Aarons G. A. James S. Monn A. R. Raghavan R. Wells R. S. Leslie L. K. (2010). Behavior problems and placement change in a national child welfare sample: A prospective study. J. Am. Acad. Child Adolesc. Psychiatry 49, 70–80. doi: 10.1016/j.jaac.2009.09.005 20215928 PMC4131764

[ref2] Achenbach T. M. (1991). Manual for the Teacher’s Report Form and 1991 Profile. Burlington: University of Vermont.

[ref3] Achenbach T. M. Rescorla L. A. (2001). Manual for the ASEBA School Age Forms & Profiles. Burlington: University of Vermont.

[ref4] Achenbach T. M. Rescorla L. A. Dias P. Ramalho V. Lima V. S. Machado B. C. Gonçalves M. (2014). Manual do sistema de avaliação empiricamente validado (ASEBA) para o período pré-escolar e escolar: Um sistema integrado de avaliação com múltiplos informadores. Braga: Psiquilibrios Edições.

[ref5] An E. M. Lee S. J. Chung I.-J. (2020). The effects of the stigma trajectory of adolescents in out-of-home care on self-esteem and antisocial behavior. Child Youth Serv. Rev. 116:105167. doi: 10.1016/j.childyouth.2020.105167

[ref6] Arpini D. M. (2003). Repensando a perspectiva institucional e a intervenção em abrigos para crianças e adolescentes. Psicologia: Ciência E Profissão 23, 70–75. doi: 10.1590/s1414-98932003000100010

[ref7] Assouline A. H. Attar-Schwartz S. (2020). Staff social support and adolescent adjustment difficulties: The moderating role of length of stay in the residential care setting. Child Youth Serv. Rev. 110:104761. doi: 10.1016/j.childyouth.2020.104761

[ref8] Attar-Schwartz S. (2009). School functioning of children in residential care: The contributions of multilevel correlates. Child Abuse Negl. 33, 429–440. doi: 10.1016/j.chiabu.2008.12.010 19589598

[ref9] Baams L. Beek T. Hille H. Zevenbergen F. C. Bos H. M. (2013). Gender nonconformity, perceived stigmatization, and psychological well-being in Dutch sexual minority youth and young adults: A mediation analysis. Arch. Sex. Behav. 42, 765–773. doi: 10.1007/s10508-012-0055-z 23358856

[ref11] Barendregt C. S. Van der Laan A. M. Bongers I. L. Van Nieuwenhuizen C. (2015). Adolescents in secure residential care: the role of active and passive coping on general well-being and self-esteem. Eur. Child Adolesc. Psychiatry 24, 845–854. doi: 10.1007/s00787-014-0629-5 25325990

[ref12] Bastiaanssen I. L. W. Delsing M. J. M. H. Kroes G. Engels R. C. M. E. Veerman J. W. (2014). Group care worker interventions and child problem behavior in residential youth care: course and bidirectional associations. Child Youth Serv. Rev. 39, 48–56. doi: 10.1016/j.childyouth.2014.01.012

[ref13] Bois J. E. Sarrazin P. G. Brustad R. J. Chanal J. P. Trouilloud D. O. (2005). Parents' appraisals, reflected appraisals, and children's self-appraisals of sport competence: A yearlong study. J. Appl. Sport Psychol. 17, 273–289. doi: 10.1080/10413200500313552

[ref14] Branje S. J. Van Aken M. A. Van Lieshout C. F. Mathijssen J. J. (2003). Personality judgments in adolescents' families: The perceiver, the target, their relationship, and the family. J. Pers. 71, 49–81. doi: 10.1111/1467-6494.t01-1-00001 12597237

[ref15] Cahill O. Holt S. Kirwan G. (2016). Keyworking in residential child care: lessons from research. Child Youth Serv. Rev. 65, 216–223. doi: 10.1016/j.childyouth.2016.04.014

[ref16] Calheiros M. M. Garrido M. V. Lopes D. Patrício J. N. (2015). Social images of residential care: how children, youth and residential care institutions are portrayed? Child Youth Serv. Rev. 55, 159–169. doi: 10.1016/j.childyouth.2015.06.004

[ref17] Calheiros M. M. Patrício J. N. Silva C. S. (2020a). Social support as a moderator of associations between youths’ perceptions of their social images and self-representations in residential care. Child Youth Serv. Rev. 119:105667. doi: 10.1016/j.childyouth.2020.105667

[ref18] Calheiros M. M. Silva C. S. Magalhães E. (2021). Child maltreatment severity questionnaire (MSQ) for professionals: development, validity, and reliability evidence. Assessment 28, 1397–1417. doi: 10.1177/1073191119890030 31793334

[ref19] Calheiros M. M. Silva C. S. Patrício J. N. (2020b). Maltreatment and youth self-representations in residential care: The moderating role of individual and placement variables. Child Youth Serv. Rev. 116:105230. doi: 10.1016/j.childyouth.2020.105230

[ref20] Campos J. Barbosa-Ducharne M. Dias P. Rodrigues S. Martins A. C. Leal M. (2019). Emotional and behavioral problems and psychosocial skills in adolescents in residential care. Child Adolesc. Soc. Work J. 36, 237–246. doi: 10.1007/s10560-018-0594-9

[ref21] Carvalho H. (2008). Análise de Multivariada de Dados Qualitativos, Utilização da Análise de Correspondências Múltiplas com o SPSS. Lisboa: Edições Sílabo.

[ref22] Charon J. M. (1985). Symbolic Interactionism: An Introduction, an Interpretation, an Integration. Englewood Cliffs, NJ: Prentice Hall.

[ref23] Cicchetti D. Lynch M. (1993). Toward an ecological/transactional model of community violence and child maltreatment: consequences for children’s development. Psychiatry 56, 96–118. doi: 10.1080/00332747.1993.11024624 8488217

[ref24] Cole D. A. (1991). Change in self-perceived competence as a function of peer and teacher evaluation. Dev. Psychol. 27, 682–688. doi: 10.1037/0012-1649.27.4.682

[ref26] Cooley C. H. (1902). Human Nature and Social Order. 1964 Edn. New York: Schribner’s.

[ref27] Corrigan P. W. Sokol K. A. Rüsch N. (2013). The impact of self-stigma and mutual help programs on the quality of life of people with serious mental illnesses. Community Ment. Health J. 49, 1–6. doi: 10.1007/s10597-011-9445-2 22038373 PMC3320674

[ref28] Crocker J. Major B. (1989). Social stigma and self-esteem: The self-protective properties of stigma. Psychol. Rev. 96, 608–630. doi: 10.1037/0033-295X.96.4.608

[ref29] Crocker J. Quinn D. M. (2000). “Social stigma and the self: meanings, situations, and self-esteem,” in The Social Psychology of Stigma. eds. Heatherton T. F. Kleck R. E. Hebl M. R. Hull J. G. (New York: Guilford Press), 153–183.

[ref115] Dishion T. J. McCord J. Poulin F. (1999). When interventions harm. Am. Psychol. 54, 755–764. doi: 10.1037/0003-066X.54.9.755, PMID: 10510665

[ref32] Gallagher S. Zahavi D. (2007). The Phenomenological Mind: An Introduction to Philosophy of Mind and Cognitive Science. New York: Routledge.

[ref116] Garrido M. V. Nunes Patrício J. Calheiros M. M. Lopes D. (2016). Comparing the social images of youth in and out of residential care. J. Community Appl. Soc. Psychol. 26, 439–455. doi: 10.1002/casp.2273

[ref33] Gearing R. Schwalbe C. MacKenzie M. Brewer K. Ibrahim R. (2014). Assessment of adolescent mental health and behavioral problems in institutional care: discrepancies between staff-reported CBCL scores and adolescent-reported YSR scores. Adm. Policy Ment. Health Ment. Health Serv. Res. 42, 279–287. doi: 10.1007/s10488-014-0568-y 24938476

[ref34] GEP/MTSS (2018) Gabinete de Estratégia e Planeamento/Ministério do Trabalho, Solidariedade e Segurança Social. Carta Social. Available at: http://www.cartasocial.pt/index2.php# (September 20, 2018).

[ref35] Gifi A. (1996). Nonlinear Multivariate Analysis. New York: Wiley.

[ref36] González-García C. Bravo A. Arruabarrena I. Martín E. Santos I. Del Valle J. F. (2017). Emotional and behavioral problems of children in residential care: screening detection and referrals to mental health services. Child Youth Serv. Rev. 73, 100–106. doi: 10.1016/j.childyouth.2016.12.011

[ref37] Goodman R. (1997). The strengths and difficulties questionnaire: A research note. J. Child Psychol. Psychiatry 38, 581–586. doi: 10.1111/j.1469-7610.1997.tb01545.x 9255702

[ref117] Goodman R. Meltzer H. Bailey V. (2003). The Strengths and difficulties questionnaire: a pilot study on the validity of the self-report version. Int. Rev. Psychiatry 15, 173–177. doi: 10.1080/0954026021000046137 12745329

[ref38] Greenacre M. (2007). Correspondence Analysis in Practice. London: Chapman & Hall/CRC.

[ref39] Greve W. Enzmann D. (2003). Self-esteem maintenance among incarcerated young males: stabilisation through accommodative processes. Int. J. Behav. Dev. 27, 12–20. doi: 10.1080/01650250143000562

[ref40] Harter S. (1999). The Construction of the Self. A Developmental Perspective. New York: Guilford Press.

[ref41] Harter S. (2015). The Construction of the Self: Developmental and Sociocultural Foundations. New York: Guilford Publication.

[ref42] Hatzenbuehler M. L. (2016). Structural stigma: research evidence and implications for psychological science. Am. Psychol. 71, 742–751. doi: 10.1037/amp0000068 27977256 PMC5172391

[ref43] Hatzenbuehler M. L. Phelan J. C. Link B. G. (2013). Stigma as a fundamental cause of population health inequalities. Am. J. Public Health 103, 813–821. doi: 10.2105/AJPH.2012.301069 23488505 PMC3682466

[ref44] Hayes A. F. (2015). An index and test of linear moderated mediation. Multivar. Behav. Res. 50, 1–22. doi: 10.1080/00273171.2014.962683 26609740

[ref45] Heflinger C. A. Simpkins C. G. Combs-Orme T. (2000). Using the CBCL to determine the clinical status of children in state custody. Child Youth Serv. Rev. 22, 55–73. doi: 10.1016/S0190-7409(99)00073-0

[ref46] Hiles D. Moss D. Thorne L. Wright J. Dallos R. (2014). “So what am I?” — multiple perspectives on young people’s experience of leaving care. Child Youth Serv. Rev. 41, 1–15. doi: 10.1016/j.childyouth.2014.03.007

[ref47] Hodas G. (2005). Empowering Direct Care Workers Who Work with Children and Youth in Institutional Care. Harrisburg: Pennsylvania Office of Mental Health and Substance Abuse Services.

[ref49] Holden M. (2009). Children and Residential Experiences (CARE): Creating Conditions for Change. Arlington, VA: Child Welfare League of America.

[ref50] Holden M. J. Anglin J. P. Nunno M. A. Izzo C. V. (2014). “Engaging the total therapeutic residential care program in a process of quality improvement: learning from the CARE model,” in Therapeutic Residential Care with Children and Youth: Identifying Promising Pathways to Evidence Based International Practice. eds. Whittaker J. K. Fernandez Del Valle J. Holmes L. (Philadelphia, PA: Jessica Kingsley), 301–315.

[ref51] Holden M. J. Sellers D. (2019). An evidence-based program model for facilitating therapeutic responses to pain-based behavior in residential care. Int. J. Child Youth Family Stud. 10, 63–80. doi: 10.18357/ijcyfs102-3201918853

[ref52] Hukkanen R. Sourander A. Bergroth L. Piha J. (1999). Psychosocial factors and adequacy of services for children in children’s homes. Eur. Child Adolesc. Psychiatry 8, 268–275. doi: 10.1007/s007870050101 10654120

[ref53] Hussey D. L. Guo S. (2002). Profile characteristics and behavioral change trajectories of young residential children. J. Child Fam. Stud. 11, 401–410. doi: 10.1023/a:1020927223517

[ref54] Ibrahim R. W. Howe D. (2011). The experience of Jordanian care leavers making the transition from residential care to adulthood: The influence of a patriarchal and collectivist culture. Child Youth Serv. Rev. 33, 2469–2474. doi: 10.1016/j.childyouth.2011.08.019

[ref55] Instituto da Segurança Social (ISS), IP. (2020). CASA 2019: Caracterização anual da situação de acolhimento das crianças e jovens. Retrieved from https://www.seg-social.pt/documents/10152/13200/Relat%C3%B3rio+CASA+2019/0bf7ca2b-d8a9-44d2-bff7-df1f111dc7ee

[ref56] Izzo C. V. Smith E. G. Sellers D. E. Holden M. J. Nunno M. A. (2020). Improving relationship quality in group care settings: The impact of implementing the CARE model. Child Youth Serv. Rev. 109:104623. doi: 10.1016/j.childyouth.2019.104623

[ref57] Jones R. Everson-Hock E. S. Papaioannou D. Guillaume L. Goyder E. Chilcott J.. (2011). Factors associated with outcomes for looked-after children and young people: a correlates review of the literature. Child Care Health Dev. 37, 613–622. doi: 10.1111/j.1365-2214.2011.01226.x 21434967 PMC3500671

[ref58] Jozefiak T. Kayed N. S. Rimehaug T. Wormdal A. K. Brubakk A. M. Wichstrøm L. (2016). Prevalence and comorbidity of mental disorders among adolescents living in residential youth care. Eur. Child Adolesc. Psychiatry 25, 33–47. doi: 10.1007/s00787-015-0700-x 25749933 PMC4698296

[ref59] Kenny D. A. Albright L. Malloy T. E. Kashy D. A. (1994). Consensus in interpersonal perception: acquaintance and the big five. Psychol. Bull. 116, 245–258. doi: 10.1037/0033-2909.116.2.245 7972592

[ref60] Kepper A. Monshouwer K. Van Dorsselaer S. Vollebergh W. (2011). Substance use by adolescents in special education and residential youth care institutions. Eur. Child Adolesc. Psychiatry 20, 311–319. doi: 10.1007/s00787-011-0176-2 21573695 PMC3098996

[ref61] Kools S. M. (1997). Adolescent identity development in foster care. Fam. Relat. 46, 263–271. doi: 10.2307/585124

[ref62] Kuznetsova T. I. (2005). Social stereotypes of the perception of graduates of Children’s homes. Russ. Educ. Soc. 47, 19–30. doi: 10.1080/10609393.2005.11056948

[ref63] Labouvie-Vief G. Chiodo L. M. Goguen L. A. Diehl M. (1995). Representations of self across the life span. Psychol. Aging 10, 404–415. doi: 10.1037/0882-7974.10.3.404 8527061

[ref64] Lanctôt N. Lemieux A. Mathys C. (2016). The value of a safe, connected social climate for adolescent girls in residential care. Resid. Treat. Child. Youth 33, 247–269. doi: 10.1080/0886571x.2016.1207218

[ref66] Law n° 142/2015 of the Assembly of the Republic (2015). Republic Diary: Series I, n° 175. Available at: https://data.dre.pt/eli/lei/142/2015/09/08/p/dre/pt/html (Accessed September 29, 2021).

[ref67] Lee B. R. Thompson R. (2008). Comparing outcomes for youth in treatment foster care and family-style group care. Child Youth Serv. Rev. 30, 746–757. doi: 10.1016/j.childyouth.2007.12.002 19122763 PMC2515489

[ref68] Lehmann S. Havik O. E. Havik T. Heiervang E. R. (2013). Mental disorders in foster children: a study of prevalence, comorbidity and risk factors. Child Adolesc. Psychiatry Ment. Health 7:39. doi: 10.1186/1753-2000-7-39 24256809 PMC3922948

[ref69] Liebling A. (1993). Suicides in young prisoners: A summary. Death Stud. 17, 381–407. doi: 10.1080/07481189308253385

[ref70] Lin X. Fang X. Liu Y. Lan J. (2009). The effect mechanism of stigma perception on mental health among migrant children in Beijing. Acta Psychol. Sin. 41, 967–979. doi: 10.3724/SP.J.1041.2009.00967

[ref71] Magalhães E. Calheiros M. M. (2017). A dual-factor model of mental health and social support: evidence with adolescents in residential care. Child Youth Serv. Rev. 79, 442–449. doi: 10.1016/j.childyouth.2017.06.041

[ref72] Magalhães E. Calheiros M. M. Antunes C. (2018). ‘I always say what I think’: a rights-based approach of young people’s psychosocial functioning in residential care. Child Indic. Res. 11, 1801–1816. doi: 10.1007/s12187-017-9511-6

[ref73] Major B. O’brien L. T. (2005). The social psychology of stigma. Annu. Rev. Psychol. 56, 393–421. doi: 10.1146/annurev.psych.56.091103.070137 15709941

[ref74] Mann M. M. Hosman C. M. Schaalma H. P. De Vries N. K. (2004). Self-esteem in a broad-spectrum approach for mental health promotion. Health Educ. Res. 19, 357–372. doi: 10.1093/her/cyg041 15199011

[ref75] Marsh H. W. Craven R. G. (2006). Reciprocal effects of self-concept and performance from a multidimensional perspective: Beyond seductive pleasure and unidimensional perspectives. Perspect. Psychol. Sci. 1, 133–163. doi: 10.1111/j.1745-6916.2006.00010.x 26151468

[ref76] Marshall G. Winter K. Turney D. (2020). Honneth and positive identity formation in residential care. Child Fam. Soc. Work 25, 733–741. doi: 10.1111/cfs.12749

[ref77] McAdams D. P. (2013). The psychological self as actor, agent, and author. Perspect. Psychol. Sci. 8, 272–295. doi: 10.1177/1745691612464657 26172971

[ref78] McMurray I. Connolly H. Preston-Shoot M. Wigley V. (2011). Shards of the old looking glass: restoring the significance of identity in promoting positive outcomes for looked-after children. Child Fam. Soc. Work 16, 210–218. doi: 10.1111/j.1365-2206.2010.00733.x

[ref79] Mead G. H. (1934). Mind, Self and Society. Vol. 11. Chicago: University of Chicago Press.

[ref80] Montserrat C. Casas F. Malo S. (2013). Delayed educational pathways and risk of social exclusion: the case of young people from public care in Spain. Eur. J. Soc. Work. 16, 6–21. doi: 10.1080/13691457.2012.722981

[ref81] Mullan C. McAlister S. Rollock F. Fitzsimons L. (2007). “Care just changes your life”: factors impacting upon the mental health of children and young people with experiences of care in Northern Ireland. Child Care Pract. 13, 417–434. doi: 10.1080/13575270701488865

[ref82] Noble-Carr D. Woodman E. (2018). Considering identity and meaning constructions for vulnerable young people. J. Adolesc. Res. 33, 672–698. doi: 10.1177/0743558416684952

[ref118] Nurra C. Pansu P. (2009). The impact of significant others’ actual appraisals on children’s self-perceptions: What about Cooley’s assumption for children? Eur. J. Psychol. Educ. 24, 247–262. doi: 10.1007/BF03173015

[ref83] O’Mara A. J. Marsh H. W. Craven R. G. Debus R. L. (2006). Do self-concept interventions make a difference? A synergistic blend of construct validation and meta-analysis. Educ. Psychol. 41, 181–206. doi: 10.1207/s15326985ep4103_4

[ref84] Pascoe E. A. Richman L. S. (2009). Perceived discrimination and health: A meta-analytic review. Psychol. Bull. 135, 531–554. doi: 10.1037/a0016059 19586161 PMC2747726

[ref85] Patrício J. N. Calheiros M. M. Martins A. C. (2016). Self-representation questionnaire for youth in residential care. Child Youth Serv. Rev. 61, 317–326. doi: 10.1016/j.childyouth.2016.01.007

[ref87] Pfeifer J. H. Masten C. L. Borofsky L. A. Dapretto M. Fuligni A. J. Lieberman M. D. (2009). Neural correlates of direct and reflected self-appraisals in adolescents and adults: when social perspective-taking informs self-perception. Child Dev. 80, 1016–1038. doi: 10.1111/j.1467-8624.2009.01314.x 19630891 PMC3229828

[ref88] Preacher K. J. Rucker D. D. Hayes A. F. (2007). Addressing moderated mediation hypotheses: theory, methods, and prescriptions. Multivar. Behav. Res. 42, 185–227. doi: 10.1080/00273170701341316 26821081

[ref89] Puhl R. M. King K. M. (2013). Weight discrimination and bullying. Best Pract. Res. Clin. Endocrinol. Metab. 27, 117–127. doi: 10.2105/ajph.2009.159491 23731874

[ref90] Richardson J. Lelliott P. (2003). Mental health of looked after children. Adv. Psychiatr. Treat. 9, 249–256. doi: 10.1192/apt.9.4.249

[ref91] Rodrigues S. P. D. L. A. (2019). A qualidade do acolhimento residencial em Portugal: Avaliação da adequação dos serviços às necessidades das crianças e jovens institucionalizados (Tese de doutoramento) [The quality of residential care in Portugal: Assessment of the adequacy of services to the needs of institutionalized children and young people PhD Dissertation]. Porto, Portugal: University of Porto.

[ref92] Rodrigues S. Barbosa-Ducharne M. del Valle J. F. (2013). The quality of residential child care in Portugal and the example of its development in Spain. Papeles del Psicólogo 34, 11–22.

[ref93] Rodrigues S. Barbosa-Ducharne M. Del Valle J. F. Campos J. (2019). Psychological adjustment of adolescents in residential care: comparative analysis of youth self-report/strengths and difficulties questionnaire. Child Adolesc. Soc. Work J. 36, 247–258. doi: 10.1007/s10560-019-00614-x

[ref119] Rodríguez A. del Valle J. F. Bravo A. (2015). Detección de problemas de salud mental en un grupo especialmente vulnerable: niños y adolescentes en acogimiento residencial. Anales de Psicología/Ann. Psychol. 31, 472–480. doi: 10.6018/analesps.31.2.182051

[ref94] Rosenberg M. Schooler C. Schoenbach C. Rosenberg F. (1995). Global self-esteem and specific self-esteem: different concepts, different outcomes. Am. Sociol. Rev. 60, 141–156. doi: 10.2307/2096350

[ref95] Rosenthal J. A. Villegas S. (2010). Living situation and placement change and children’s behavior. Child Youth Serv. Rev. 32, 1648–1655. doi: 10.1016/j.childyouth.2010.07.003

[ref96] Ruch G. Turney D. Ward A. (2010). Relationship Based Social Work. London: Jessica Kingsley Publishers.

[ref97] Rutter M. (2000). Children in substitute care: Some conceptual considerations and research implications. Child Youth Serv. Rev. 22, 685–703. doi: 10.1016/s0190-7409(00)00116-x

[ref120] Ryan J. P. Marshall J. M. Herz D. Hernandez P. M. (2008). Juvenile delinquency in child welfare: Investigating group home effects. Child. Youth Serv. Rev 30, 1088–1099. doi: 10.1016/j.childyouth.2008.02.004

[ref99] Schmid M. Goldbeck L. Nuetzel J. Fegert J. M. (2008). Prevalence of mental disorders among adolescents in German youth welfare institutions. Child Adolesc. Psychiatry Ment. Health 2:2. doi: 10.1186/1753-2000-2-2 18226213 PMC2262059

[ref101] Shen J. (2016). Principles and applications of multilevel modeling in human resource management research. Hum. Resour. Manag. 55, 951–965. doi: 10.1002/hrm.21666

[ref102] Silva C. S. Calheiros M. M. (2020). Maltreatment experiences and psychopathology in children and adolescents: The intervening role of domain-specific self-representations moderated by age. Child Abuse Negl. 99:104255. doi: 10.1016/j.chiabu.2019.104255 31791007

[ref103] Silva C. S. Calheiros M. M. Carvalho H. Magalhães E. (2021). Organizational social context and psychopathology of youth in residential care: the intervening role of youth-caregiver relationship quality. Appl. Psychol. 1–23. doi: 10.1111/apps.12339

[ref104] Simkiss D. (2013). “Looked-after children and young people,” in Annual Report of the Chief Medical Officer 2012. Our Children Deserve Better: Prevention Pays. ed. Davis D. S. (UK: Department of Health).

[ref105] Simsek Z. Erol N. Öztop D. Münir K. (2007). Prevalence and predictors of emotional and behavioral problems reported by teachers among institutionally reared children and adolescents in Turkish orphanages compared with community controls. Child Youth Serv. Rev. 29, 883–899. doi: 10.1016/j.childyouth.2007.01.004 25520538 PMC4266372

[ref106] Smith M. Cameron C. Reimer D. (2017). From attachment to recognition for children in care. Br. J. Soc. Work. 47, 1606–1623. doi: 10.1093/bjsw/bcx096

[ref107] Sulimani-Aidan Y. (2016). In between formal and informal: staff and youth relationships in care and after leaving care. Child Youth Serv. Rev. 67, 43–49. doi: 10.1016/j.childyouth.2016.05.025

[ref108] Tarren-Sweeney M. Vetere A. (2013). “Establishing the need for mental health services for children and young people in care, and those who are subsequently adopted,” in Mental Health Services for Vulnerable Children and Young People: Supporting Children Who Are, or Have Been, in Foster Care. eds. Tarren-Sweeney M. Vetere A. (London: Routledge).

[ref109] Taussig H. N. (2002). Risk behaviors in maltreated youth placed in foster care: A longitudinal study of protective and vulnerability factors. Child Abuse Negl. 26, 1179–1199. doi: 10.1016/s0145-2134(02)00391-5 12398855

[ref110] Turner R. Whitehead C. (2008). How collective representations can change the structure of the brain. J. Conscious. Stud. 15, 43–57.

[ref111] Villagrana M. Guillen C. Macedo V. Lee S. (2018). Perceived self-stigma in the utilization of mental health services in foster care and post foster care among foster care alumni. Child Youth Serv. Rev. 85, 26–34. doi: 10.1016/j.childyouth.2017.10.040

[ref112] Vojak C. (2009). Choosing language: social service framing and social justice. Br. J. Soc. Work. 39, 936–949. doi: 10.1093/bjsw/bcm144

[ref113] Waniel A. Besser A. Priel B. (2006). Mother and self-representations: investigating associations with symptomatic behavior and academic competence in middle childhood. J. Pers. 74, 223–266. doi: 10.1111/j.1467-6494.2005.00374.x 16451231

[ref114] Whittaker J. K. Holmes L. Del Valle J. F. Ainsworth F. Andreassen T. Anglin J.. (2016). Therapeutic residential care for children and youth: A consensus statement of the international work group on therapeutic residential care. Resid. Treat. Child. Youth 33, 89–106. doi: 10.1080/0886571X.2016.1215755

[ref121] Zhao X. Lynch J. G. Jr. Chen Q. (2010). Reconsidering Baron and Kenny: Myths and truths about mediation analysis. J. Consum. Res 37, 197–206. doi: 10.1086/651257

